# The Role of Diet in Multiple Sclerosis Onset: A Prospective Study Using UK Biobank

**DOI:** 10.3390/nu16111746

**Published:** 2024-06-02

**Authors:** Camilla Barbero Mazzucca, Lorenza Scotti, Cristoforo Comi, Domizia Vecchio, Annalisa Chiocchetti, Giuseppe Cappellano

**Affiliations:** 1 Department of Health Sciences, Interdisciplinary Research Center of Autoimmune Diseases-IRCAD, Università del Piemonte Orientale, 28100 Novara, Italy; camilla.barbero@uniupo.it (C.B.M.); giuseppe.cappellano@med.uniupo.it (G.C.); 2 Center for Translational Research on Autoimmune and Allergic Diseases, University of Piemonte Orientale, 28100 Novara, Italy; 3 Department of Translational Medicine, University of Piemonte Orientale, 28100 Novara, Italy; lorenza.scotti@uniupo.it (L.S.); cristoforo.comi@med.uniupo.it (C.C.); domizia.vecchio@uniupo.it (D.V.); 4 MS Centre, Azienda Ospedaliero-Universitaria Maggiore della Carità, 28100 Novara, Italy; 5 Department of Neurology, University of Piemonte Orientale, 28100 Novara, Italy; 6 Neurology Unit, S. Andrea Hospital, Department of Translational Medicine, University of Piemonte Orientale, 13100 Vercelli, Italy

**Keywords:** multiple sclerosis, diet, autoimmune diseases, UK Biobank

## Abstract

Multiple sclerosis (MS) is a debilitating autoimmune condition primarily affecting young adults, and its rise is evident globally. Despite this, its precise etiology remains elusive. Both genetic and environmental factors contribute to MS susceptibility; however, the link between diet and MS lacks substantial evidence due to limited large-scale studies. We exploited the UK Biobank resources to explore the nexus between diet, lifestyle, and MS risk. The dietary and lifestyle habits of MS incident cases, derived from a general food frequency questionnaire (FFQ) completed by all participants at study enrollment, were compared to those of subjects who did not develop MS during the follow-up. Our findings suggest the protective role of moderate oily fish consumption and weekly alcohol intake. Furthermore, by analyzing food intake data obtained through 24 h recall, completed by a subset of participants, we found a protective, though non-significant, trend of an increased adherence to the Mediterranean diet (MD). These findings, derived from the analysis of the UK Biobank and representing an unprecedented approach for this inquiry, warrant further exploration and integration in future research.

## 1. Introduction

Multiple sclerosis (MS) is an autoimmune chronic inflammatory disease most commonly affecting young adults between 20 and 40 years old, but possibly arising at any age. It is characterized by autoimmune central nervous system (CNS) lesions that can lead to severe physical or cognitive disability, which include paralysis, sensory disturbances, lack of coordination, and visual impairments [1]. Different disease subtypes have been recognized (clinically isolated syndrome, relapsing-remitting, primary progressive, and secondary progressive MS) [2] and recent studies have proposed more precise classifications (i.e., different MS phenotypes) [3].

The estimated incidence of MS is between 8 and 11 new cases diagnosed each year in England per 100,000 population, with women outnumbering men two to one [4,5].

Although the etiology and pathogenesis of MS remain unclear, the current evidence suggests that MS is a multifactorial disease in which both genetic predisposition and environmental factors may play a role. Contact with infectious agents, such as Epstein–Barr virus (EBV), exposure to ultraviolet Blight (UVB), obesity, and smoking have been indicated as possible risk factors [2]. In a previous review, we discussed the importance of considering the influence of gut health on CNS; specifically, the connection between CNS and the enteric nervous system (ENS) and the existence of a gut–brain axis supports the importance of considering diet as an important modulator of gut homeostasis and, consequently, of CNS health [6]. The ”leaky gut” condition, induced by the frequent consumption of food additives and often coexisting with dysbiosis, has been associated with an increased risk of AD onset. Specifically, a pro-inflammatory gut environment may lead to an altered communication with CNS, increasing neuroinflammation and MS risk [2]. Interestingly, the liver recently emerged as an additional distant organ influencing brain health, with the liver–brain axis also being studied in the context of MS. This evidence encourages the study of diet as a modifiable risk factor for the neurological disease [7].

In a recent case–control study performed on the UK Biobank cohort, the authors demonstrated that living a healthy lifestyle (i.e., avoiding smoking, following a healthy diet, having a body mass index (BMI) <30 kg/m^2^, and meeting the physical activity guidelines) is inversely associated with MS prevalence [8]. More recently, a cross-sectional study conducted on people living with MS and healthy controls revealed that the dietary quality of MS patients was greater than that of healthy controls. Precisely, MS patients showed a greater consumption of foods that are commonly regarded as healthy, such as fish and vegetables; surprisingly, their intake appeared to be highly intercorrelated and positively associated with disability outcomes [9]. An ecological correlation between fiber intake and MS disability outcome was observed also in another cross-sectional assessment [10]; however, these findings could also be the result of a reverse causality effect, a bias that could exist in cross-sectional research to explain why patients may begin eating healthier once they are aware of their illness status.

On the other hand, other studies indicate a protective effect of whole grains [11] and vegetable intake [12] due to their high content of flavonoids, which have been indicated as powerful in improving MS in preclinical studies [13]. Regarding fish, other studies indicate its long-term protective effect [14,15] against the risk of developing MS, possibly due to its vitamin D content, although this hypothesis has been disproved by other evidence indicating that low fish consumption slightly increases MS risk by other means than by affecting vitamin D status [16]. Vitamin D deficiency is recognized as a risk factor for MS [17]; however, dairy products, which are often fortified for vitamin D, have been positively associated with MS risk [18], and abnormal T-cell responses against multiple cow-milk proteins have been found in children with autoimmune diseases (type one diabetes, MS) and CNS injuries [19]. On the other hand, a case–control study, which retrospectively collected nutrition information from MS patients and healthy controls, unveiled a protective role of vegetables, fruits, seafood, dairy, red and white meat, and nuts [20].

Overall, the relationship between single foods and MS risk is still under debate, and the reasons are heterogeneous. Firstly, differences in genetic background, microbiota composition, and the dietary habits of different populations may render it more challenging to obtain results that are valid for the overall population. Moreover, the reduced effect size of single foods, if considered individually, on clinical outcomes makes it difficult to evaluate their impact in cross-sectional studies or reduced cohorts, and the possibility of reverse causality, specifically due to study design, cannot be excluded. Notably, there is a paucity of large-scale prospective studies regarding these different dietary exposures that are focused on the general population [21]. In the present work, we used the data collected in the UK Biobank, one of the biggest cohort studies available to date, to investigate the possible link of diet with the onset of MS.

## 2. Methods

### 2.1. Cohort Selection

This is a prospective cohort study based on data collected from the UK Biobank, and is covered by generic ethical approval from the NHS Research Ethics Committee (Ref. 11/NW/0382) for UK Biobank research. The use of UK Biobank data, originally collected between 2017 and 2019, was approved within project 91267 on 10 November 2022.

The UK Biobank is one of the largest publicly available health-focused resources, aiming at understanding the impact of environmental factors, genetics, and lifestyle upon a broad array of health outcomes.

The study cohort consisted of 502,507 subjects aged 40–69 years, living in England, Wales, or Scotland, who were invited by formal mail to participate in the UK Biobank cohort study in 2006 (pilot phase) and 2007–2010 (main phase). More than 500,000 subjects attended the first assessment visit to UK Biobank assessment centers and were asked to complete a touchscreen questionnaire aimed at collecting information regarding sociodemographic characteristics, lifestyle habits, medical history, and dietary habits, among others. For the main study, subjects were excluded based on the following exclusion criteria: prevalent cases (affected by MS before cohort entry); subjects who withdrew their consent to participate in the study or had missing information on all dietary factors; and subjects who self-reported MS at any time during the study period, since the diagnosis date was unknown and only the estimated year of MS onset was available. The presence of MS was identified as a hospital admission with a primary or secondary diagnosis of MS (International Classification of Disease (ICD)-9 code 340 or ICD-10 codes G35) or self-reported diagnosis of MS (code 1464).

### 2.2. Dietary Data Collection

At the study’s baseline (2006), all study participants completed a food frequency questionnaire (FFQ) during the assessment visit, reporting their usual intake over the last year of 29 different food groups and alcohol. Specifically, participants were asked about their daily intake of cooked vegetables, salad/raw vegetables, fresh, dried fruit, coffee (decaffeinated or caffeinated) and tea, as well as their weekly consumption of oily fish, other fish, processed meats, poultry, beef, lamb, pork, cheese, salt added to food, tea, water, milk, spreads, bread, and breakfast cereals [18]. For daily intake, subjects could report the exact number of tablespoons/serving consumed, whereas for weekly intake, they could choose one of the following options: never, less than once a week, once a week, 2–4 times a week, 5–6 times a week, or once or more daily.

A subsample of enrolled subjects additionally participated in an online interview that focused on diet during the previous 24 h (24 h recall). The interview was sent via email to every participant (about 320,000 people), asking them to complete it four times between February 2011 and April 2012, an approximate annual span. It was based on a thorough investigation of 200 distinct foods and beverages consumed over the previous 24 h. Since it automatically calculated the energy and nutritional contents of the reported food items, we were able to evaluate the impact of single micro- or macronutrient intake on the disease risk. To be consistent with the FFQ data, we considered the information from the online 24 h recall questionnaire collected at the first repeat of the assessment visit.

The dietary intake by Oxford WebQ was also exploited to calculate the Mediterranean diet (MDS) score, as was previously performed by Livingstone KM et al. in the context of cardiovascular diseases [19].

### 2.3. Outcome Ascertainment

Information on subjects’ hospitalization was retrieved through record linkage with NHS hospital in-patient data from hospital episode statistics in England, the Scottish Morbidity Records, and the Patient Episode Database for Wales. Outcome ascertainment: MS definition.

Incident MS cases were selected based on subjects’ hospitalization data through record linkage with NHS in-patient data from hospital episode statistics in England, the Scottish Morbidity Records, and the Patient Episode Database for Wales.

Subjects hospitalized with primary or secondary diagnoses of MS identified by ICD-10 codes G35 were classified as events. Subjects were followed from the date of the first assessment visit, during which nutritional information was collected, until the earliest date on which one of the following events took place: (i) hospitalization with a diagnosis of MS, (ii) death, (iii) migration, or (iv) end of follow-up (30 September 2021).

### 2.4. Statistical Analysis

Descriptive statistics were utilized to summarize the main characteristics of the subjects included in the study and their dietary habits. Categorical variables were reported as absolute frequencies and percentages. Univariable and multivariable Cox proportional hazard models were fitted to assess to estimate the hazard ratios (HRs) and the corresponding 95% confidence intervals (95%CI) for the association between dietary factors and risk of MS. The lowest consumption level was considered as the reference category. Multivariable models were adjusted for age, sex, study area, Townsend deprivation index, smoking, BMI, moderate/vigorous physical activity, alcohol consumption, and comorbidities (Type II diabetes, hypertension, hyperlipidemia, and other autoimmune disease). The test for trends was also performed for the multivariable model. The same models were applied to estimate the relationship between adherence to the MD and the risk of MS. Regarding nutrients, the intake was classified according to the tertiles of the distribution, and MDS according to quartiles.

## 3. Results

### 3.1. Lifestyle as a Risk Factor for MS in UK Biobank

**Figure 1:**
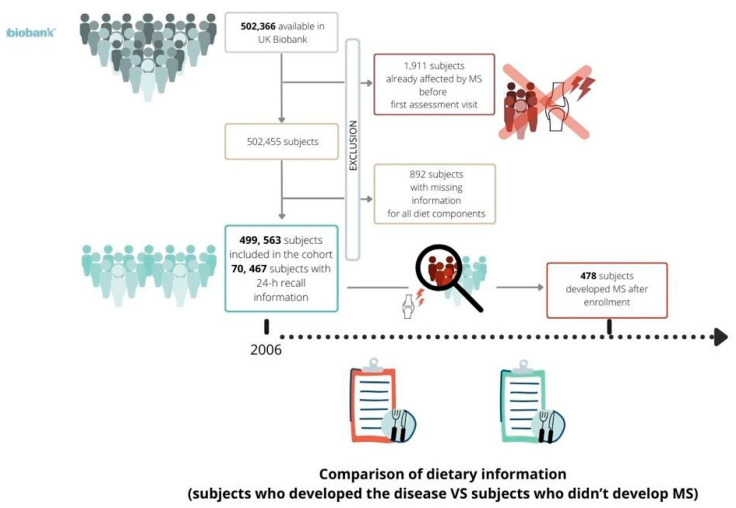
Flow chart of exclusion criteria which led to the definition of the final cohort.

From the initial cohort included in the UK Biobank, consisting of 502,366 subjects (people who retracted their consent to participate already excluded), 1911 were excluded because of prevalent MS, while a further 892 subjects were removed from the analysis because of missing information for all diet components. Once the exclusion criteria had been applied (Figure 1), the final cohort consisted of 499,563 subjects; among them, 478 subjects were identified as incident MS cases. Among the 207,144 participants who completed the Oxford WebQs, only 70,467 participants also had 24 h recall nutritional data available; among them, 67 subjects developed MS after enrolment.

Cohort members accumulated 614,1445.67 years of long-term follow-up (12.29 years per subject as an average) and generated 478 events of MS, leading to an incidence rate of 7.78 MS events per 100,000-person year.

Table 1 shows the distribution of the cohort members’ primary characteristics overall and by event status, as well as the associated HR and 95% confidence interval (CI) from the univariable Cox regression model. All considered variables are associated with MS onset. As expected, male subjects had a lower risk of developing MS (HR 0.593, 95%CI 0.490–0.717). Compared to younger subjects, those between 50 and 60 years old or older than 60 had a decreased risk of developing MS, with HRs of 0.691 (95%CI 0.559–0.854) and 0.549 (CI 0.439–0.686), respectively.

Current smokers showed an increased disease risk (HR 1.922, CI 1.471–2.511) compared to never smokers, while being past smokers did not modify the risk of developing MS.

Practicing moderate or vigorous physical activity seemed to reduce the disease risk for all the considered frequency levels compared to a sedentary lifestyle; specifically, a frequency of 1–3 days a week resulted to be the most protective (HR 0.515 95%CI 0.398–0.666), followed by 7 times/week (HR 0.538, 95%CI 0.399–0.725) and 4–6 times/week (HR 0.570, 95%CI 0.439–0.479), while BMI did not affect the risk of developing MS.

Regarding comorbidities, diabetes, hypertension, and hypercholesterolemia did not seem to be associated with the risk of MS onset, while subjects affected by other autoimmune diseases had a risk of developing MS 2.052 times higher compared to subjects without autoimmune diseases (95%CI 1.061–3.967).

### 3.2. Oily Fish and Alcohol Consumption as Protective Factors for MS

Oily fish intake was associated with a decreased MS risk compared to no consumption; specifically, eating oily fish once a week showed a protective effect (aHR 0.642, 95%CI 0.480–0.859), as did consuming it two or more times per week (aHR 0.666, 95%CI 0.474–0.934). We also found a statistically significant trend regarding oily fish intake (*p*-value 0.0035).

**Figure 2:**
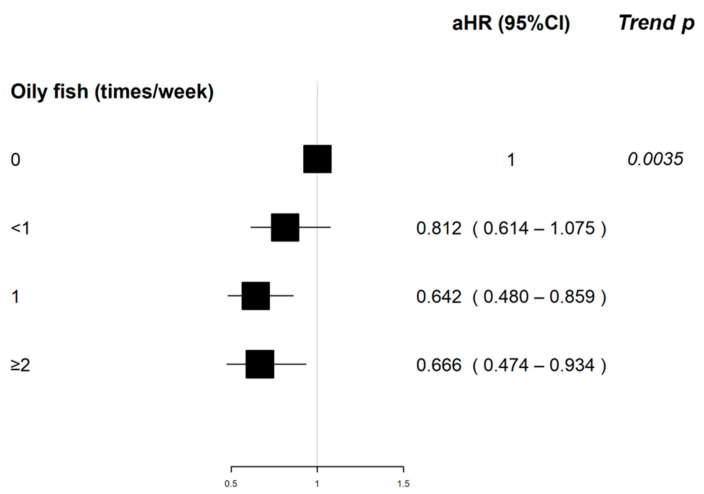
Adjusted HR and corresponding 95% CI for the association between selected food groups and MS and *p*-value of the trend test.

The frequency of alcohol consumption was inversely associated with MS risk, with weekly consumption showing an HR of 0.799 (95%CI 0.648–0.984) compared to monthly consumption. Table 2 describes the distribution of the frequency of consumption of different food groups, the unadjusted and adjusted HR, and the corresponding 95%CI for the association between food intake and MS risk; the same result is depicted in a forest plot (Figure 2).

### 3.3. Adherence to Mediterranean Diet and MS risk

When broadening the perspective to complex dietary patterns, a non-significant trend emerged, suggesting a positive correlation between the adherence to MD (increase of one point in MDS score) and disease risk (Table 3).

## 4. Discussion

The multifaceted nature of MS remains unclear in terms of etiopathogenesis. The goal of the present study is to assess the role of diet and other lifestyle factors in disease risk by exploiting one of the largest currently available databases, the UK Biobank, with which we previously developed a pipeline to study the role of environmental factors in rheumatoid arthritis onset [22]. The available evidence regarding the role of lifestyle factors in modifying the risk of developing the disease is still limited and occasionally contradictory. The causal relationship between smoking and the risk of developing MS has been demonstrated by quality studies, suggesting that quitting smoking could prevent at least 13% of cases of MS. Smoking increases lung inflammation acting at the cellular level of the immune system, resulting in the development of proinflammatory cytokines, which can in turn favor autoimmune attacks against CNS antigens in the lungs [23]. Our data show an increased risk of developing MS in current smokers, but not in past smokers [24]. This finding was not expected, since childhood and adolescence have been indicated as delicate times in which environmental risk factors for MS tend to exert their most powerful effects [25]. However, a case–control study indicated that both current and past smokers have an increased odds of developing MS [26].

Moreover, the increased risk among current smokers found in our study could be even higher among subjects with previous Epstein–Barr infection, vitamin D deficiency, or human leukocyte antigen (HLA) DR15*1501, given the known interaction effect with these environmental and genetic factors [27].

Childhood and adolescent obesity has been proposed as a risk factor for MS, and genetic determinants for obesity have been associated with an increased risk of MS. The underlying cause may be related to the state of low-grade chronic inflammation; the increased levels of leptin, which determine the production of proinflammatory cytokines; and, finally, the decreased bioavailability of vitamin D associated with obesity status [28]. The first comprehensive exploration into the link between obesity and MS risk occurred within the Nurses’ Health Study (NHS) I and II. It revealed that women with a BMI of 30 kg/m^2^ or higher at age 18 faced an elevated risk of developing the disease. However, no correlation was identified between the baseline BMI and MS risk in the study [29]. Similarly, we did not observe any association with BMI; thus, this can be explained by the fact that we considered the BMI at the enrollment in the UK Biobank (baseline) and not weight status during childhood or adolescence, which was not available in the data set.

Alongside body mass index and smoking, physical activity has also been studied as a modifiable risk factor for MS. Studies with different designs (case–control [30], observational [31], and Mendelian randomization [32] studies) have confirmed our results, indicating a protective role of moderate/vigorous physical activity.

To our knowledge, this is the first time that a protective effect of moderate fish consumption has been observed; moreover, from our study, eating oily fish once a week resulted to be slightly more protective than a more frequent intake. Since, overall, the available evidence to date are mainly coming from case–control studies, which often stratify subjects into high and low fish consumers, our results may suggest a finer dose–response effect.

According to our study, moderate fatty fish intake reduces the risk of developing MS. These results add novel insights into the previously suggested preventive effect of this food. Indeed, a meta-analysis published in 2020 stated that fatty fish intake during adolescence or later in life is inversely correlated with MS risk, and may be most advantageous for persons who reside in areas with lower levels of solar exposure to make up for the reduced sun exposure and vitamin D synthesis [14]. A previous case–control study restricted the protective effect to fatty fish, for which an intake level from one to seven times per week decreased MS risk compared to lower consumption. According to the authors, the protective effect of fatty fish is most likely attributable to vitamin D, higher concentrations of which in serum have been found among eaters of fatty fish compared to eaters of lean fish [15]. Another case–control study found that increased fish consumption reduced the incidence of central nervous system (CNS) demyelination; among different fish types, an increased intake of up to two servings per week of canned fish, which is predominantly oily, led to a 40% reduction in the chance of developing CNS demyelination, while no association emerged with grilled or fried fish [33].

As anticipated, the immunomodulatory functions of vitamin D likely mediate the preventive effect of fatty fish. Nonetheless, fatty fish is also a good source of Omega-3 polyunsaturated fatty acids (PUFA) [34]. PUFA exhibit powerful immunomodulatory proprieties and, according to preclinical and clinical intervention studies, may be helpful in the prevention of AD and inflammatory diseases; particularly, eicosapentaenoic acid (EPA) and docosahexaenoic acid (DHA) from fish oil are more biologically potent than alpha-linolenic acid (ALA), which is mainly found in vegetable foods [35]. Moreover, long-chain omega-3s, such as DHA, showed neuroprotective effects in CNS [36], where they also exert a structural role [37]. According to a systematic review conducted on the topic of EPA, DHA, DPA (docosapentaenoic acid), and fish oil supplementation in the amount of 4 g per day have beneficial effects in terms of reducing the relapse rate and inflammation in MS patients, improving their quality of life overall [15].

On the other hand, the presence of proinflammatory compounds in fish may attenuate its protective effect; consistently, in a previous study, we demonstrated the protective effect of moderate fish consumption on rheumatoid arthritis onset in the UK Biobank cohort and we argued concerning the harmful effects of TMAO (Trimethylamine N-oxide) and fish contaminants, such as Bisphenol A (BPA), which can counter-balance the protective role of omega-3s and vitamin D [22].

Only a few studies have prospectively examined the impact of alcohol use on MS risk, often leading to inconsistent results. The effects of ethanol on immune system regulation have been dissected in a recent review, which suggested that acute and moderate alcohol consumption have a suppressing effect on the immune system and its responses to pathogens, while chronic alcohol consumption also suppresses general immune system activity, but increases the response to the pathogens [38]. One prospective study did not find any impact [39] of alcohol on MS risk, while another report based on two case–control studies suggested an inverse, dose-dependent association between MS and alcohol intake; in one of the two analyzed cohorts, a protective role of both spirits and wine for MS onset emerged. These studies, however, may be susceptible to reverse causation and recall bias due to their design [40]. Our work supports an inverse association between weekly alcohol consumption, although without a significant trend, and MS risk, adopting a prospective, and thus more solid, approach with a larger cohort. Unfortunately, the structure of the touchscreen FFQ administered at study enrollment did not allow for a deeper analysis of the type of alcoholic beverages that contribute to disease risk modification. Thus, this evaluation should be performed in future studies to confirm our observations and explore the side effects of alcohol consumption; specifically, data from more specific nutritional surveys, as the OxfordWebQ, completed by a subset of UK Biobank participants, can be analyzed to gather more specific insights and different perspectives, given that 24 h recall data refer to actual and not usual consumption.

It is often difficult to identify possible correlations between individual foods and clinical outcomes; in this study, we prospectively examined a large cohort of subjects enrolled in the UK Biobank. However, the most recent research in nutritional immunology focuses on the role of complex nutritional patterns, rather than on single foods/nutrients, in chronic disease risk. Specifically, the MD pattern has been thoroughly studied in the context of non-communicable disease prevention [41,42,43,44,45]. Concerning AD and MS prevention, MD has all the credentials to be looked at as a preventive dietary pattern, and evidence is available regarding its possible immune-modulatory and antioxidant effects on SNC [6,46]. We thus deemed it appropriate to estimate the adherence to MD by exploiting the Oxford WEBQ, as was previously carried out by Livingstone KM et al. [47], since the assessment-visit FFQ was too generic for this purpose. The MDS adherence score is a validated MD adherence score commonly used in clinical practice [48,49]. MDS was calculated for both cases and controls, revealing a non-significative trend according to which an increase of 1 point was associated with an aHR lower than 1. Despite the suggested preventive effect of MD on MS risk, to our knowledge, only case–control studies have been conducted on the topic, indicating a protective effect of MD on disease risk [50,51]. For this reason, our work provides a wider, population-based perspective. However, the major limitation of using the Oxford WebQ is the fact that the 24 h recall may not be the most appropriate tool for usual dietary pattern assessment. Moreover, the reduced case number may imply a reduction in the statistical power of the analysis. Despite this, we deemed it valuable to carry out this investigation to provide a new perspective besides the commonly used case–control approach, which is susceptible to different types of biases. Moreover, given the present dynamic context in which adherence to MD could be influenced by the increasing trend of the Western diet (WD), it would be interesting to evaluate the influence of complex dietary patterns on clinical outcomes by exploiting new tools, thus overcoming the limits of single adherence scores and allowing us to estimate the adherence to these opposite dietary patterns in a combined way [46].

Similarly, the exploitation of Oxford WebQ for the dissection of the role of different food sub-types (e.g., distinguishing different types of alcoholic beverages when evaluating the effect of alcohol intake) could be considered as a different approach to study the role of diet in MS onset in future studies. Since this different type of dietary evaluation has been conducted at subsequent time points after enrollment, Oxford WebQ may be of interest for further long-term evaluations.

## 5. Conclusions

In the present study, we exploited the UK Biobank database to investigate the role of diet in MS onset using a prospective and multi-faceted approach. The FFQ completed by all enrolled subjects at the assessment visit was used to determine whether single food items were associated with disease onset, revealing a protective role of moderate fish and alcohol consumption. According to our knowledge, this is the first study that has prospectively investigated the role of single nutrients in MS onset in the UK Biobank cohort. Moreover, by means of a validated approach, we exploited the 24 h recall data to estimate the adherence to the MD, providing new insights into the role of complex dietary patterns, instead of single foods, in disease onset. These data provide a basis for more specific studies addressing the role of diet in MS onset, which may help to build evidence-based indications for MS prevention and management. Moreover, given the heterogeneity of disease subtypes and the existence of different MS “phenotypes”, the application of this pipeline on other study cohorts, in which the number of incident cases is enough to permit further stratifications, may lead to new insights useful for personalizing dietary approaches in the context of precision nutrition.

## Figures and Tables

**Table 1:** Distribution of the main characteristics of the cohort members, hazard ratios (HRs), and corresponding 95% confidence intervals (CIs) for the association with multiple sclerosis (MS).

		Multiple Sclerosis (MS)		
	Overall	No	Yes	
	N = 499,563	N = 499,085	N = 478	HR (95%CI)
	N (%)	N (%)	N (%)	
Sex				
Females	271,445 (54.34)	271,124 (54.32)	321 (67.15)	1
Males	228,118 (45.66)	227,961 (45.68)	157 (32.85)	0.593 (0.490–0.717)
Age				
≤50	131,231 (26.27)	131,053 (26.26)	178 (37.24)	1
(50–60]	176,141 (35.26)	175,978 (35.26)	163 (34.1)	0.691 (0.559–0.854)
>60	192,190 (28.47	192,053 (38.48)	137 (28.66)	0.549 (0.439–0.686)
Missing	1	1	0	
Area				
England	443,228 (88.72)	442,792 (88.72)	436 (91.21)	1
Scotland	35,636 (7.13)	35,606 (7.13)	30 (6.28)	0.821 (0.567–1.189)
Wales	20,699 (4.14)	20,687 (4.14)	12 (2.51)	0.573 (0.323–1.017)
Townsend deprivation index				
Q1	124,914 (25.04)	124,794 (25.04)	120 (25.1)	0.950 (0.739–1.222)
Q2	124,786 (25.01)	124,661 (25.01)	125 (26.15)	0.996 (0.776–1.277)
Q3	124,790 (25.01)	124,680 (25.01)	110 (23.01)	0.882 (0.682–1.140)
Q4	124,455 (24.94)	124,332 (24.94)	123 (25.73)	1
Missing	618	618	0	
Smoking				
Current smokers	52,656 (10.58)	52,576 (10.58)	80 (16.84)	1.922 (1.471–2.511)
Partial smokers	244,700 (49.19)	244,469 (49.19)	231 (48.63)	1.164 (0.953–1.422)
Never smokers	200,117 (40.23)	199,953 (40.23)	164 (34.53)	1
Missing	2090	2087	3	
BMI categories (kg/m^2^)				
Normal (<25)	164,235 (33.05)	164,075 (33.05)	160 (34.04)	1
Overweight (25.0–29.9)	211,185 (42.49)	210,993 (42.49)	192 (40.85)	0.936 (0.759–1.154)
Obese (≥30.0)	121,564 (24.46)	121,446 (24.46)	118 (25.11)	1.008 (0.795–1.279)
Missing	2579	2571	8	
Moderate/vigorous physical activity (days/week)				
0	61,672 (12.63)	61,576 (12.62)	96 (20.87)	1
1–3	181,318 (37.13)	181,170 (37.14)	148 (32.17)	0.515 (0.398–0.666)
4–6	153,205 (31.37)	153,067 (31.38)	138 (30.00)	0.570 (0.439–0.739)
7	92,123 (18.87)	92,045 (18.87)	78 (16.96)	0.538 (0.399–0.725)
Missing	11,245	11,227	18	
Type II diabetes				
No	491,278 (98.34)	490,807 (98.34)	471 (98.54)	1
Yes	8285 (1.66)	8278 (1.66)	7 (1.46)	0.922 (0.437–1.945)
Hypertension				
No	368,342 (73.73)	367,986 (73.73)	356 (74.48)	1
Yes	131,221 (26.27)	131,099 (26.27)	122 (25.52)	0.983 (0.800–1.207)
Hypercholesterolemia				
No	474,798 (95.04)	474,337 (95.04)	461 (96.44)	1
Yes	24,765 (4.96)	24,748 (4.96)	17 (3.56)	0.713 (0.440–1.157)
Other autoimmune disease				
No	494,944 (99.07)	494,475 (99.08)	469 (98.12)	1
Yes	4619 (0.92)	4610 (0.92)	9 (1.88)	2.052 (1.061–3.967)

**Table 2:** Distribution of the diet components, unadjusted and adjusted hazard ratios (HRs), and corresponding 95% CIs for the association with MS and *p*-value of the trend test.

	Consumption				
Cooked Vegetables (tbs/day)	≤1	2	≥3		Trend *p*-Value
N events/non events	100/90,239	153/163,064	216/239,146		
HR (95%CI)	1	0.844 (0.656–1.086)	0.816 (0.644–1.035)		
aHR (95%CI)	1	0.922 (0.709–1.199)	0.896 (0.697–1.151)		0.4126
Salad/vegetables (tbs/day)	≤1	2	3	≥4	
N events/non events	222/218,622	99/119,214	67/69,848	82/84,508	
HR (95%CI)	1	0.816 (0.644–1.034)	0.942 (0.717–1.238)	0.954 (0.741–1.229)	
aHR (95%CI)	1	0.798 (0.624–1.02)	0.909 (0.684–1.207)	0.908 (0.697–1.183)	0.4239
Fresh fruit (serving/day)	≤1	2	3	≥4	
N events/non events	162/176,419	136/140,184	84/99,644	94/80,527	
HR (95%CI)	1	1.05 (0.836–1.319)	0.91 (0.699–1.184)	1.263 (0.98–1.628)	
aHR (95%CI)	1	1.098 (0.868–1.39)	0.888 (0.672–1.173)	1.271 (0.972–1.662)	0.3000
Oily fish (times/week)	0	<1	1	≥2	
N events/non events	76/54,519	176/164,067	153/187,604	70/89,747	
HR (95%CI)	1	0.767 (0.586–1.004)	0.584 (0.444–0.769)	0.561 (0.406–0.777)	
aHR (95%CI)	1	0.812 (0.614–1.075)	0.642 (0.480–0.859)	0.666 (0.474–0.934)	0.0035
Other fish (times/week)	<1	1	≥2		
N events/non events	164/167,463	226/247,211	83/81,624		
HR (95%CI)	1	0.934 (0.764–1.142)	1.040 (0.799–1.355)		
aHR (95%CI)	1	1.012 (0.822–1.246)	1.081 (0.821–1.422)		0.6178
Processed meat (times/week)	0	<1	1	≥2	
N events/non events	48/46,470	159/151,541	130/145,323	140/154,440	
HR (95%CI)	1	1.014 (0.734–1.400)	0.868 (0.624–1.209)	0.886 (0.639–1.230)	
aHR (95%CI)	1	1.037 (0.741–1.451)	1.001 (0.709–1.413)	1.061 (0.749–1.503)	0.7992
Chicken, turkey, or other poultry (times/week)	<1	1	≥2		
N events/non events	75/79,115	172/178,778	231/240,041		
HR (95%CI)	1	1.01 (0.770–1.325)	1.005 (0.775–1.305)		
aHR (95%CI)	1	1.091 (0.822–1.448)	0.998 (0.759–1.312)		0.7523
Beef (times/week)	0	<1	1	≥2	
N events/non events	59/55,240	218/226,008	141/157,969	56/57,477	
HR (95%CI)	1	0.903 (0.677–1.204)	0.836 (0.617–1.132)	0.913 (0.633–1.315)	
aHR (95%CI)	1	1.038 (0.765–1.408)	0.990 (0.716–1.369)	1.095 (0.743–1.614)	0.8508
Lamb/mutton (times/week)	0	<1	≥1		
N events/non events	86/88,140	275/280,367	112/127,027		
HR (95%CI)	1	1.006 (0.789–1.281)	0.912 (0.689–1.208)		
aHR (95%CI)	1	1.116 (0.865–1.439)	1.130 (0.841–1.517)		0.4425
Pork (times/week)	0	<1	≥1		
N events/non events	89/85,953	261/281,408	124/128,364		
HR (95%CI)	1	0.895 (0.704–1.139)	0.942 (0.717–1.236)		
aHR (95%CI)	1	0.988 (0.767–1.271)	1.079 (0.81–1.437)		0.5560
Cheese (times/week)	<1	1	≥2		
N events/non events	96/97,784	97/104,308	272/283,564		
HR (95%CI)	1	0.945 (0.713–1.253)	0.974 (0.772–1.229)		
aHR (95%CI)	1	1.004 (0.749–1.345)	1.049 (0.821–1.339)		0.6644
Bread (slices/week)	≤7	8–13	≥14		
N events/non events	182/157,754	177/196,101	107/135,406		
HR (95%CI)	1	0.785 (0.638–0.965)	0.690 (0.543–0.876)		
aHR (95%CI)	1	0.889 (0.717–1.101)	0.831 (0.641–1.078)		0.1456
Breakfast cereals (bowls/week)	≤3	4–6	≥7		
N events/non events	171/175,626	136/132,735	168/188,563		
HR (95%CI)	1	1.043 (0.833–1.307)	0.911 (0.736–1.127)		
aHR (95%CI)	1	1.059 (0.838–1.338)	0.970 (0.774–1.214)		0.7929
Tea (cups/day)	0	≤2	3–4	≥5	
N events/non events	77/73,051	116/129,031	125/145,825	158/149,911	
HR (95%CI)	1	0.849 (0.636–1.132)	0.809 (0.609–1.075)	0.999 (0.761–1.312)	
aHR (95%CI)	1	0.851 (0.631–1.148)	0.889 (0.665–1.190)	1.056 (0.798–1.397)	0.3868
Alcohol consumption (drinks/time)	≤3 times a month	1–4 times week	Daily or almost daily		
N events/non events	182/153,520	216/243,663	80/101,297		
HR (95%CI)	1	0.739 (0.607–0.900)	0.665 (0.511–0.865)		
aHR (95%CI)	1	0.799 (0.648–0.984)	0.764 (0.578–1.010)		0.9860

**Table 3:** Association between adherence to MD, calculated through MDS score, and MS risk. Unadjusted and adjusted HRs and corresponding 95% CIs for association with MS.

MDS	HR (95%CI)	aHR (95%CI)
Increase of 1 point	0.970 (0.826–1.139)	0.958 (0.811–1.133)
MDS		
≤2	1	1
3	0.801 (0.410–1.566)	0.824 (0.421–1.612)
4	1.183 (0.634–2.205)	1.236 (0.660–2.314)
>4	0.921 (0.464–1.828)	0.844 (0.405–1.756)

## Data Availability

The original contributions presented in the study are included in the article, further inquiries can be directed to the corresponding author.

## References

[1] Naseri A., Nasiri E., Sahraian M.A., Daneshvar S., Talebi M. (2021). Clinical Features of Late-Onset Multiple Sclerosis: A Systematic Review and Meta-Analysis. Mult. Scler. Relat. Disord..

[2] Ghasemi N., Razavi S., Nikzad E. (2017). Multiple Sclerosis: Pathogenesis, Symptoms, Diagnoses and Cell-Based Therapy. Cell J..

[3] Pitt D., Lo C.H., Gauthier S.A., Hickman R.A., Longbrake E., Airas L.M., Mao-Draayer Y., Riley C., De Jager P.L., Wesley S. (2022). Toward Precision Phenotyping of Multiple Sclerosis. Neurol. Neuroimmunol. Neuroinflamm..

[4] Steinman L. (2001). Multiple Sclerosis: A Two-Stage Disease. Nat. Immunol..

[5] Multiple Sclerosis: Prevalence, Incidence and Smoking Status—Data Briefing—GOV.UK. https://www.gov.uk/government/publications/multiple-sclerosis-prevalence-incidence-and-smoking-status/multiple-sclerosis-prevalence-incidence-and-smoking-status-data-briefing#:~:text=Conclusions,-The%20purpose%20of&text=The%20estimate%20for%20the%20prevalence,cases%20per%20100%2C000%20population%20respectively.

[6] Barbero Mazzucca C., Cappellano G., Chiocchetti A. (2023). Nutrition, Immunity and Aging: Current Scenario and Future Perspectives in Neurodegenerative Diseases. CNS Neurol. Disord. Drug Targets.

[7] D’Mello C., Swain M.G. (2011). Liver-Brain Inflammation Axis. Am. J. Physiol. Gastrointest. Liver Physiol..

[8] Veronese N., Yang L., Piccio L., Smith L., Firth J., Marx W., Giannelli G., Caruso M.G., Cisternino A.M., Notarnicola M. (2020). Adherence to a Healthy Lifestyle and Multiple Sclerosis: A Case–Control Study from the UK Biobank. Nutr. Neurosci..

[9] Guillet C., Rastmanesh R., Ruggieri S., De Giglio L., Felicetti F., Tommasin S., Petracca M., Gurreri F., Ianniello A., Nistri R. (2022). Eating Hubs in Multiple Sclerosis: Exploring the Relationship Between Mediterranean Diet and Disability Status in Italy. Front. Nutr..

[10] Coe S., Tektonidis T.G., Coverdale C., Penny S., Collett J., Chu B.T.Y., Izadi H., Middleton R., Dawes H. (2021). A Cross Sectional Assessment of Nutrient Intake and the Association of the Inflammatory Properties of Nutrients and Foods with Symptom Severity in a Large Cohort from the UK Multiple Sclerosis Registry. Nutr. Res..

[11] Fitzgerald K.C., Tyry T., Salter A., Cofield S.S., Cutter G., Fox R., Marrie R.A. (2018). Diet Quality Is Associated with Disability and Symptom Severity in Multiple Sclerosis. Neurology.

[12] Hadgkiss E.J., Jelinek G.A., Weiland T.J., Pereira N.G., Marck C.H., van der Meer D.M. (2015). The Association of Diet with Quality of Life, Disability, and Relapse Rate in an International Sample of People with Multiple Sclerosis. Nutr. Neurosci..

[13] Bayat P., Farshchi M., Yousefian M., Mahmoudi M., Yazdian-Robati R. (2021). Flavonoids, the Compounds with Anti-Inflammatory and Immunomodulatory Properties, as Promising Tools in Multiple Sclerosis (MS) Therapy: A Systematic Review of Preclinical Evidence. Int. Immunopharmacol..

[14] Rezaeizadeh H., Mohammadpour Z., Bitarafan S., Harirchian M.H., Ghadimi M., Homayon I.A. (2022). Dietary Fish Intake and the Risk of Multiple Sclerosis: A Systematic Review and Meta-Analysis of Observational Studies. Nutr. Neurosci..

[15] Bäärnhielm M., Olsson T., Alfredsson L. (2014). Fatty Fish Intake Is Associated with Decreased Occurrence of Multiple Sclerosis. Mult. Scler..

[16] Hedström A.K., Olsson T., Kockum I., Hillert J., Alfredsson L. (2020). Low Fish Consumption Is Associated with a Small Increased Risk of MS. Neurol. (R) Neuroimmunol. Neuroinflamm..

[17] Ascherio A., Munger K.L., Simon K.C. (2010). Vitamin D and Multiple Sclerosis. Lancet Neurol..

[18] Munger K.L., Chitnis T., Frazier A.L., Giovannucci E., Spiegelman D., Ascherio A. (2011). Dietary Intake of Vitamin D during Adolescence and Risk of Multiple Sclerosis. J. Neurol..

[19] Banwell B., Bar-Or A., Cheung R., Kennedy J., Krupp L.B., Becker D.J., Dosch H.M. (2008). Abnormal T-Cell Reactivities in Childhood Inflammatory Demyelinating Disease and Type 1 Diabetes. Ann. Neurol..

[20] Sharifi M.H., Keshani P., Salehi A., Jaladat A.M., Mirzaei Z., Nikseresht A. (2021). Association between Multiple Sclerosis and Dietary Patterns Based on the Traditional Concept of Food Nature: A Case-Control Study in Iran. BMC Neurol..

[21] Katz Sand I. (2018). The Role of Diet in Multiple Sclerosis: Mechanistic Connections and Current Evidence. Curr. Nutr. Rep..

[22] Mazzucca C.B., Scotti L., Cappellano G., Barone-Adesi F., Chiocchetti A. (2022). Nutrition and Rheumatoid Arthritis Onset: A Prospective Analysis Using the UK Biobank. Nutrients.

[23] Nishanth K., Tariq E., Nzvere F.P., Miqdad M., Cancarevic I. (2020). Role of Smoking in the Pathogenesis of Multiple Sclerosis: A Review Article. Cureus.

[24] Manouchehrinia A., Huang J., Hillert J., Alfredsson L., Olsson T., Kockum I., Constantinescu C.S. (2022). Smoking Attributable Risk in Multiple Sclerosis. Front. Immunol..

[25] Ascherio A., Munger K.L. (2007). Environmental Risk Factors for Multiple Sclerosis. Part II: Noninfectious Factors. Ann. Neurol..

[26] Maghzi A.H., Etemadifar M., Heshmat-Ghahdarijani K., Moradi V., Nonahal S., Ghorbani A., Minagar A. (2011). Cigarette Smoking and the Risk of Multiple Sclerosis: A Sibling Case-Control Study in Isfahan, Iran. Neuroepidemiology.

[27] Wingerchuk D.M. (2012). Smoking: Effects on Multiple Sclerosis Susceptibility and Disease Progression. Ther. Adv. Neurol. Disord..

[28] Olsson T., Barcellos L.F., Alfredsson L. (2017). Interactions between Genetic, Lifestyle and Environmental Risk Factors for Multiple Sclerosis. Nat. Rev. Neurol..

[29] Gianfrancesco M.A., Barcellos L.F. (2016). Obesity and Multiple Sclerosis Susceptibility: A Review. J. Neurol. Neuromed..

[30] Wesnes K., Myhr K.M., Riise T., Cortese M., Pugliatti M., Boström I., Landtblom A.M., Wolfson C., Bjørnevik K. (2018). Physical Activity Is Associated with a Decreased Multiple Sclerosis Risk: The EnvIMS Study. Mult. Scler..

[31] Dorans K.S., Massa J., Chitnis T., Ascherio A., Munger K.L. (2016). Physical Activity and the Incidence of Multiple Sclerosis. Neurology.

[32] Li C., Lin J., Yang T., Xiao Y., Jiang Q., Shang H. (2022). Physical Activity and Risk of Multiple Sclerosis: A Mendelian Randomization Study. Front. Immunol..

[33] Black L.J., Zhao Y., Peng Y.C., Sherriff J.L., Lucas R.M., van der Mei I., Pereira G., Chapman C., Coulthard A., Dear K. (2020). Higher Fish Consumption and Lower Risk of Central Nervous System Demyelination. Eur. J. Clin. Nutr..

[34] Langer-Gould A., Black L.J., Waubant E., Smith J.B., Wu J., Gonzales E.G., Shao X., Koebnick C., Lucas R.M., Xiang A. (2020). Seafood, Fatty Acid Biosynthesis Genes, and Multiple Sclerosis Susceptibility. Mult. Scler..

[35] Simopoulos A.P. (2002). Omega-3 fatty acids in inflammation and autoimmune diseases. J. Am. Coll. Nutr..

[36] Siegert E., Paul F., Rothe M., Weylandt K.H. (2017). The Effect of Omega-3 Fatty Acids on Central Nervous System Remyelination in Fat-1 Mice. BMC Neurosci..

[37] Agostoni C. (2008). Role of Long-Chain Polyunsaturated Fatty Acids in the First Year of Life. J. Pediatr. Gastroenterol. Nutr..

[38] Fahim M., Rafiee Zadeh A., Shoureshi P., Ghadimi K., Cheshmavar M., Sheikhinia N., Afzali M. (2020). Review Article Alcohol and Multiple Sclerosis: An Immune System-Based Review. Int. J. Physiol. Pathophysiol. Pharmacol..

[39] Massa J., O’Reilly E.J., Munger K.L., Ascherio A. (2013). Caffeine and Alcohol Intakes Have No Association with Risk of Multiple Sclerosis. Mult. Scler..

[40] Hedström A.K., Hillert J., Olsson T., Alfredsson L. (2014). Alcohol as a Modifiable Lifestyle Factor Affecting Multiple Sclerosis Risk. JAMA Neurol..

[41] Martínez-González M.A., Salas-Salvadó J., Estruch R., Corella D., Fitó M., Ros E. (2015). Benefits of the Mediterranean Diet: Insights From the PREDIMED Study. Prog. Cardiovasc. Dis..

[42] Esposito K., Maiorino M.I., Ceriello A., Giugliano D. (2010). Prevention and Control of Type 2 Diabetes by Mediterranean Diet: A Systematic Review. Diabetes Res. Clin. Pr..

[43] Gardener H., Caunca M.R. (2018). Mediterranean Diet in Preventing Neurodegenerative Diseases. Curr. Nutr. Rep..

[44] Romagnolo D.F., Selmin O.I. (2017). Mediterranean Diet and Prevention of Chronic Diseases. Nutr. Today.

[45] Shannon O.M., Ashor A.W., Scialo F., Saretzki G., Martin-Ruiz C., Lara J., Matu J., Griffiths A., Robinson N., Lillà L. (2021). Mediterranean Diet and the Hallmarks of Ageing. Eur. J. Clin. Nutr..

[46] Mazzucca C.B., Raineri D., Cappellano G., Chiocchetti A. (2021). How to Tackle the Relationship between Autoimmune Diseases and Diet: Well Begun Is Half-Done. Nutrients.

[47] Livingstone K.M., Abbott G., Bowe S.J., Ward J., Milte C., Mcnaughton S.A. (2021). Diet Quality Indices, Genetic Risk and Risk of Cardiovascular Disease and Mortality: A Longitudinal Analysis of 77 004 UK Biobank Participants. BMJ Open.

[48] Jonas B.A., Greenberg P.L. (2015). MDS Prognostic Scoring Systems—Past, Present, and Future. Best. Pr. Res. Clin. Haematol..

[49] Panagiotakos D.B., Pitsavos C., Arvaniti F., Stefanadis C. (2007). Adherence to the Mediterranean Food Pattern Predicts the Prevalence of Hypertension, Hypercholesterolemia, Diabetes and Obesity, among Healthy Adults; the Accuracy of the MedDietScore. Prev. Med..

[50] Sedaghat F., Jessri M., Behrooz M., Mirghotbi M., Rashidkhani B. (2016). Mediterranean Diet Adherence and Risk of Multiple Sclerosis: A Case-Control Study. Asia Pac. J. Clin. Nutr..

[51] Alfredsson L., Olsson T., Hedström A.K. (2023). Inverse Association between Mediterranean Diet and Risk of Multiple Sclerosis. Mult. Scler..

